# Preparation and Characterization of Porous Scaffolds Based on Poly(3-hydroxybutyrate) and Poly(3-hydroxybutyrate-*co*-3-hydroxyvalerate)

**DOI:** 10.3390/life11090935

**Published:** 2021-09-08

**Authors:** Asiyah Esmail, João R. Pereira, Chantal Sevrin, Christian Grandfils, Ugur Deneb Menda, Elvira Fortunato, Abel Oliva, Filomena Freitas

**Affiliations:** 1 Associate Laboratory i4HB—Institute for Health and Bioeconomy, School of Science and Technology, NOVA University Lisbon, 2819-516 Caparica, Portugal; a.esmail@campus.fct.unl.pt (A.E.); jra.pereira@campus.fct.unl.pt (J.R.P.); 2 UCIBIO—Applied Molecular Biosciences Unit, Department of Chemistry, School of Science and Technology, NOVA University Lisbon, 2819-516 Caparica, Portugal; 3 ITQB NOVA—Instituto de Tecnologia Química e Biológica António Xavier, NOVA University Lisbon, 2780-157 Oeiras, Portugal; oliva@itqb.unl.pt; 4 iBET, Instituto de Biologia Experimental e Tecnológica, 2780-157 Oeiras, Portugal; 5 CEIB—Interfaculty Research Centre of Biomaterials, University of Liège, B-4000 Liège, Belgium; csevrin@uliege.be (C.S.); c.grandfils@uliege.be (C.G.); 6 CENIMAT/i3N, Department of Materials Science, Nova School of Sciences and Technology, Nova University Lisbon, 2819-516 Caparica, Portugal; u.menda@fct.unl.pt (U.D.M.); emf@fct.unl.pt (E.F.)

**Keywords:** Poly(3-hydroxybutyrate), P(3HB), Poly(3-hydroxybutyrate-*co*-3-hydroxyvalerate, P(3HB-*co*-3HV), porous scaffold, salt leaching, emulsion templating

## Abstract

Poly(hydroxyalkanoates) (PHAs) with different material properties, namely, the homopolymer poly(3-hydroxybutyrate), P(3HB), and the copolymer poly(3-hydroxybutyrate-*co*-3-hydroxyvalerate, P(3HB-*co*-3HV), with a 3HV of 25 wt.%, were used for the preparation of porous biopolymeric scaffolds. Solvent casting with particulate leaching (SCPL) and emulsion templating were evaluated to process these biopolymers in porous scaffolds. SCPL scaffolds were highly hydrophilic (>170% swelling in water) but fragile, probably due to the increase of the polymer’s polydispersity index and its high porosity (>50%). In contrast, the emulsion templating technique resulted in scaffolds with a good compromise between porosity (27–49% porosity) and hydrophilicity (>30% water swelling) and without impairing their mechanical properties (3.18–3.35 MPa tensile strength and 0.07–0.11 MPa Young’s Modulus). These specifications are in the same range compared to other polymer-based scaffolds developed for tissue engineering. P(3HB-*co*-3HV) displayed the best overall properties, namely, lower crystallinity (11.3%) and higher flexibility (14.8% elongation at break. Our findings highlight the potency of our natural biopolyesters for the future development of novel porous scaffolds in tissue engineering, thanks also to their safety and biodegradability.

## 1. Introduction

Poly(hydroxyalkanoates) (PHAs) are a family of biopolyesters synthesized and accumulated intracellularly as carbon and energy reserves by many bacteria and plants [1]. Poly(3-hydroxybutyrate), P(3HB), is the most extensively studied PHA. It is resistant to hydrolytic degradation, optically pure and highly crystalline (above 70%), due to the stereoregularity that grants it rigidity [2]. Poly(3-hydroxybutyrate-*co*-3-hydroxyvalerate, P(3HB-*co*-3HV) is one of the most recognized copolymers. The incorporation of 3-hydroxyvalerate (3HV) monomers into the macromolecule grants the copolymer improved material properties, namely, lower crystallinity and lower melting temperature, as well as a reduction of its stiffness and toughness compared to the homopolymer P(3HB) [3]. Particularly, the mechanical properties of P(3HB-*co*-3HV) are strongly affected by the molar fraction of 3HV. Compared to P(3HB), the copolymer is more flexible and ductile, displaying increased elongation at breaking (50%) and decreased Young’s modulus (0.7–2.9 GPa) [2,4].

PHAs are among the most studied biopolymers for scaffold manufacturing, having been tested for the development of tissue-engineered bone, cartilage, ligament, skin, vascular tissues, neural tissues and skeletal muscles [5]. These biopolymers exhibit properties similar to conventional plastics. However, unlike fossil-based polymers that degrade slowly and continue to accumulate at alarming rates, PHAs are produced from renewable sources and are degraded to environmentally friendly constituents [6]. Moreover, these polymers and by-products have been disclosed as biocompatible on several biological models, a feature that broadens their scope of applications [7,8]. 

Scaffolds for tissue engineering are attractive 3D microenvironments enabling animal cell colonization and proliferation. For this purpose, the porous structures should have 90% porosity and must have interconnected pores, with a pore dimension of approximately 20–150 mm (for soft tissue healing). Such porous structures allow cells to interact within the 3D space, thus filling voids, bridging gaps and creating a 3D mass of cells [9,10]. Although very porous, these scaffolds should also fulfil mechanical properties to support cell growth and enhance posttraumatic organ mobilization. Mechanical strength is an essential parameter to avoid deformation of the solid structure caused by cellular loading or scaffold handling. Another relevant feature that impacts cell proliferation and differentiation is the stiffness (given by the Young’s Modulus) of the scaffold structure as cells respond to it through different mechanisms such as the activation of ion channels or protein unfolding [11]. 

A common method for 3D porous scaffold fabrication relies on emulsion templating. This technique involves the mixing of an immiscible liquid with a polymer solution, creating an emulsion that can lead, after solvent elimination, to homogenous 3D scaffolds made from interconnected pores and characterized by high porous volume [12,13]. Another widely used method is particulate leaching, which encompasses the casting of a polymer around particulates of a leachable porogen (e.g., salt particles, sugar, or paraffin spheres). A network of interconnected pores is formed upon the leaching of porogen [14].

In this work, solvent casting with particulate leaching (SCPL) and emulsion templating were compared for the fabrication of 3D porous scaffolds based on P(3HB) and P(3HB-*co*-3HV) produced by the bacterium *Cupriavidus necator* DSM428, a well-known PHA producer. The resulting scaffolds were deeply characterized in terms of morphology, crystallinity, thermal and mechanical properties, porosity, swelling and wettability in water.

## 2. Materials and Methods

### 2.1. Biopolymers

P(3HB) and P(3HB-*co*-3HV) with a 3HV content of 25 wt.% were obtained by cultivation of the bacterium *Cupriavidus necator* DSM 428 with used cooking oil as carbon source, as described by Cruz et al. [15]. Briefly, batch cultivation for P(3HB) production was carried out by cultivation in a 10 L bioreactor (BioStat B-Plus, Sartorius, Germany), and a starting volume of 8 L. A 10% (*v/v*) inoculum (800 mL) was used. The temperature and the pH were controlled at 30 ± 0.1 °C and 6.8 ± 0.05, respectively. The pH was controlled by the automatic addition of 5 M NaOH or 2 M HCl. A constant air flow rate (4 SLPM, standard liters per minute) was kept during the cultivation run, and the dissolved oxygen concentration (DO) was controlled at 30% of the air saturation by automatically adjusting the stirrer speed between 300 and 1200 rpm. For the production of the copolymer, levulinic acid was used as co-substrate, as described by Wang et al. [16]. The assay was performed under the same conditions as for P(3HB) production. However, after 21 h of cultivation, levulinic acid (300 g/L) was fed at a rate of 5 mL/h. The biopolymers were recovered from the lyophilized biomass by Soxhlet extraction with chloroform, and purified by precipitation in ice-cold ethanol, as described by Meneses et al. [17].

### 2.2. Scaffolds Fabrication

#### 2.2.1. Cast Films

**Figure 1:**
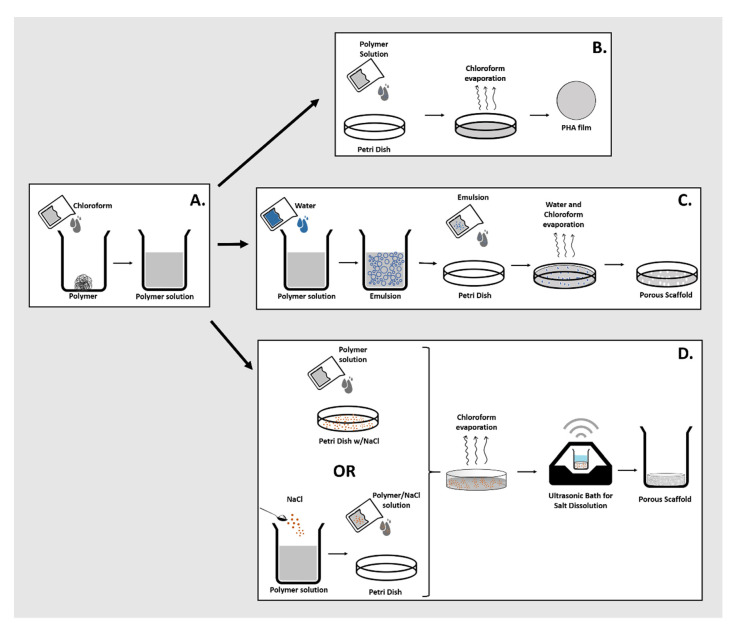
Schematic representation of PHA scaffolds preparation methodologies: (**A**) polymer dissolution; (**B**) solution casting and solvent evaporation (cast film); (**C**) emulsion templating; and (**D**) solvent-casting with particulate leaching.

The biopolymers (400 mg) were dissolved in chloroform (10 mL) (Sigma-Aldrich), under stirring, at room temperature for 24 h (Figure 1A). PHA films were obtained by casting the polymer solution into glass petri dishes (with a diameter of 5 cm) and placing them in a desiccator for 48 h, at room temperature, for slow solvent evaporation (Figure 1B). 

#### 2.2.2. Emulsion-Templated Scaffolds

The emulsion-templated scaffolds were prepared by mixing 10 mL of the polymer solution in chloroform (40 g L^−1^) with 1, 1.5, 2 and 2.5 mL of deionized water (2:20, 3:20, 4:20 and 5:20 ratios, *v/v*). The resulting mixtures were shaken manually for 1 min until an emulsion was formed. The emulsions were immediately transferred to a glass petri dish (5 cm diameter) and placed in a desiccator for 48 h, at room temperature, for slow solvents evaporation (Figure 1C).

#### 2.2.3. Particulate-Leached Scaffolds

For particulate-leached scaffolds preparation, 10 mL of the polymer solution (40 g L^−1^) was either poured onto a glass petri dish containing a layer of sodium chloride particles (106 µm in diameter) (Sigma-Aldrich, Darmstadt, Germany) or first mixed with sodium chloride particles and then poured onto a petri dish (Figure 1D). Afterwards, the solvent was evaporated in a desiccator kept at room temperature, for 48 h, in a fume hood. The particulate leaching was performed at room temperature by immersing the scaffolds in 100 mL deionized water and subjecting them to ultrasounds (Bandelin Sonorex Digitec, Berlin, Germany) to promote salt dissolution. The procedure was repeated three times for 15 min, and the conductivity of the washing water was measured to evaluate the salt’s elimination (Mettler Toledo Fiveeasy conductivity) (Figure 1D). The resulting scaffold was dried at 30 °C, in an oven, until constant weight.

### 2.3. Characterization

#### 2.3.1. Morphological Characterization

The scaffolds’ morphology was assessed by scanning electron microscopy (SEM). The samples were prepared for observation by freezing in liquid nitrogen, followed by the fracturing of the scaffolds to obtain smaller pieces with clean cross-section cuts. The samples were analyzed using a bench scanning electron microscope (TM3030 Plus + Quantax 70, Hitachi, Japan) with an acceleration voltage of 15 kV. Images of the surface and cross-section were obtained. SEM images were processed by ImageJ.

#### 2.3.2. Biopolymers’ Molecular Mass Distribution

Number and weight average molecular weights, Mn and Mw, respectively, as well as the polydispersity index (PDI), were determined by size exclusion chromatography (SEC), as described by Rebocho et al. [18]. A Waters Millenium SEC apparatus with support SEC: PLgel 5 µm Guard (Polymer Laboratories; 50 mm × 7.5 mm, PLgel 5 µm 104 Å; Polymer Lab-oratories; 300 mm × 7.5 mm, PLgel 5 μm 500 Å; Polymer Laboratories; 300 mm × 7.5 mm) was used. The analysis was performed at a temperature of equilibration of 30 °C, with a flow rate of 1 mL min^−1^, degassing and chloroform as the mobile phase. The apparatus was equipped with a solvent delivery system composed of a model 510 pump, a Rheodyne injector and a refractive index detector (Waters 2410). 

#### 2.3.3. Thermal Analysis

Thermogravimetric Analysis (TGA) was performed using a thermogravimetric equipment Labsys EVO (Setaram Instrumentation, Viroflay, France). Samples were placed in aluminum crucibles and analyzed between 25 and 500 °C, at 10 °C min^−1^. The thermal degradation temperature (T_deg_, °C) corresponds to the temperature value obtained for the maximum decreasing peak of the sample mass. Differential scanning calorimetry (DSC) analysis was done with a differential scanning calorimeter DSC25 Discovery Series (TA Instruments, Wilmington, DE, USA) equipped with a cooling system System 90 (TA Instruments, USA). The samples were placed in aluminum crucibles and analyzed between −90 and 200 °C, with heating and cooling speeds of 10 °C min^−1^, in N_2_ atmosphere (three cycles were performed). The glass transition temperature (T_g_, °C) was taken as the midpoint of the heat flux step, and the melting temperature (T_m_, °C) was determined at the minimum of the endothermic peak. The crystallinity (X_c_, %) was estimated as the ratio between melting enthalpy (ΔH_m_, J g^−1^) associated with the detected melting peak and the melting enthalpy of 100% crystalline P(3HB), estimated as 146 J g^−1^ [19].

#### 2.3.4. Water Contact Angle

The scaffolds’ water contact angle was determined by the sessile drop method. A square with dimensions of 1 cm × 1 cm of each scaffold was cut and attached to the surface analyses of the goniometer (CAM 101, KSV Instruments Ltd., Helsinki, Finland). A drop of distilled water was manually deposited on the samples’ surface with a small syringe. The software acquired ten images per sample, and the tangent of each drop was determined by fitting its shape to a known mathematical function. Multiple replicates were performed, and the mean angle was determined. All images were acquired by CAM2008 (KSV Instruments Ltd., Helsinki, Finland).

#### 2.3.5. Swelling in Water

Scaffold samples with a size of 1.0 cm^2^ were weighted, and their thickness was measured with a micrometer (Elcometer, England). The samples were immersed in 15 mL deionized water, in a closed vial, and kept at 30 °C for 24 h. The swelling degree in terms of mass of the samples was calculated with Equation (1):

(1) $$\mathrm{Swelling}\;\mathrm{Degree}\;(\%)=\frac{X2-X1}{X1}\;\times \;100$$

where X1 and X2 are, respectively, initial and final mass (g) of the samples measured at a different time. The samples thickness after immersion was also measured.

#### 2.3.6. Porosity

The porosity of the prepared scaffolds was determined as described by Kumar et al. [20]. Briefly, the scaffolds were immersed in absolute ethanol (15 mL) and kept for 5 min with brief evacuation/repressurization cycles to ensure the penetration of ethanol into the pores. The samples were weighed before and after immersion. The porosity was calculated using Equation (2):

(2) $$\mathrm{Porosity}\;(\%)=\frac{W2-W1}{\mathrm{pV}1}\;\times \;100$$

where W1 and W2 indicate the weight of the scaffolds before and after immersion, respectively; V1 is the volume before immersion; and p is the density of ethanol. Triplicate determinations were performed.

#### 2.3.7. Mechanical Properties

The scaffolds were cut into rectangular-shaped strips (~30 mm × 15 mm), and their average thickness was measured. Tensile tests were performed at ambient temperature (22 °C) using a texture analyzer (Food Technology Corporation, Kent, UK) equipped with a 50 N load cell, as described by Pereira et al. [21]. The strips were attached to tensile grips A/TG and stretched with a crosshead speed of a 100 mm/min in tension mode until breaking. The stiffness of the membranes was determined by measuring the Young’s modulus (MPa), determined as the slope of the linear initial section of the stress-strain curve. The tensile stress at breaking (MPa) was calculated as the ratio of the maximum force to the films initial cross-sectional area. The elongation (strain) at breaking (%) was determined as the ratio of the extension of the sample upon rupture by the initial gage length. Four replicas were analyzed for each scaffold.

## 3. Results

### 3.1. Biopolymers Characterization

The homopolymer P(3HB) had an M_w_ of 5.2 × 10^5^ Da and a PDI of 1.80 (Table 1). Its glass transition (T_g_) and melting temperatures (T_m_) were 2.85 and 176 °C, respectively. The biopolymer’s thermal degradation temperature (T_deg_) was 293 °C, with a crystallization temperature (T_C_) of 39 °C and corresponding cold crystallization enthalpy of 4.4 J g^−1^. The biopolymer presented a melting enthalpy of 76.5 J g^−1^ and a crystallinity degree of 52.4% (Table 1).

The copolymer P(3HB-*co*-3HV), which had a 3HV content of 25 wt.%, had M_w_ and PDI values (5.6 × 10^5^ Da and 1.60, respectively) similar to those of the homopolymer. Its T_m_ and T_deg_ were also similar (171 and 292 °C, respectively), but the copolymer was characterized by higher crystallization temperature (56 °C) and enthalpy (34.7 J g^−1^) values, and lower T_g_ (0.74 °C), melting enthalpy (34.5 J g^−1^) and crystallinity (23.6%) values, due to the reduction in stereoregularity induced by the 3HV repetitive unit in the polymer chain [3].

### 3.2. P(3HB) and P(3HB-co-3HV) Cast Films 

**Figure 2:**
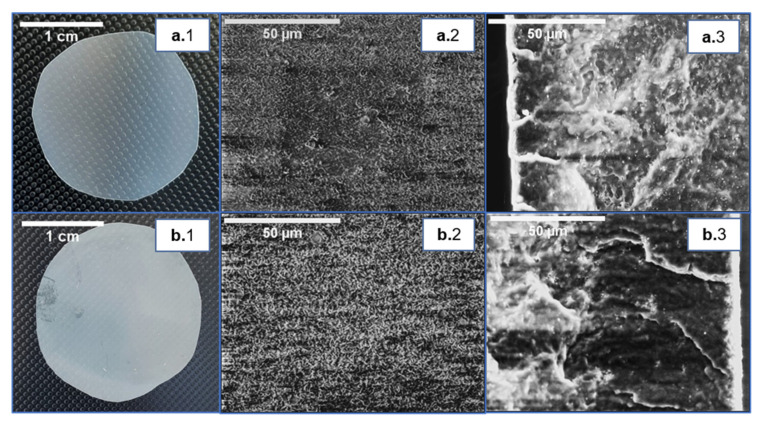
Cast films prepared from P(3HB) (**a.1**) and P(3HB-*co*-3HV) (**b.1**), and images of their surface (**center images**, (**a.2**) and (**b.2**), respectively) and cross-section (**right images**, (**a.3**) and (**b.3**), respectively) obtained by scanning electron microscopy (SEM) analysis.

The cast films were obtained from 4 wt.% biopolymer solutions in chloroform, by promoting slow solvent evaporation in a saturated chloroform atmosphere. The procedure resulted in homogenous films (Figure 2). The P(3HB) films had a thickness of 0.16 ± 0.01 mm (Table 2) and were translucent (Figure 2(a.1)). In contrast, the P(3HB-*co*-3HV) films were 0.19 ± 0.02 mm thick and presented an opaque white color (Figure 2(b.1); Table 2). Moreover, the copolymer’s cast films were more flexible than those of the homopolymer.

SEM analysis highlighted that both films had irregular surfaces (Figure 2(a.2,b.2)), exhibiting a degree of rugosity that can be linked to the semicrystalline nature and spherulite size of the polymers [22]. The films’ cross-section also revealed an irregular structure and cracks (Figure 2(a.3,b.3)), but no significant porosity across the films could be observed, which could result from the slow evaporation rate imposed during their drying. Similar morphology was reported for P(3HB) and P(3HB-*co*-3HV) cast films [23,24,25].

The P(3HB) and P(3HB-*co*-3HV) cast films presented similar water contact angles, 81.0° ± 0.8 and 78.0° ± 0.4, respectively (Table 2), indicating a comparable, non-wetting behavior. Similar values were reported for P(3HB) (63–91°) and P(3HB-*co*-3HV) (75–95°) cast films [8,13,25,26,27,28]. The films exhibited no significant change in mass or volume after water immersion. These results indicate that both films offer a low affinity with water, which agrees with the SEM imaging that revealed no noticeable porosity (Figure 2). 

### 3.3. Preparation of Porous P(3HB)- and P(3HB-co-3HV) Scaffolds

#### 3.3.1. Solution Casting with Particulate Leaching (SCPL)

The SCPL procedure was tested to prepare porous scaffolds of P(3HB) and P(3HB-*co*-3HV). Two approaches for porogen (NaCl) incorporation were tested, namely, (1) mixing the polymer solution with NaCl particles, and (2) pouring the polymer solution over a layer of this porogen (Figure 1D). 

**Figure 3:**
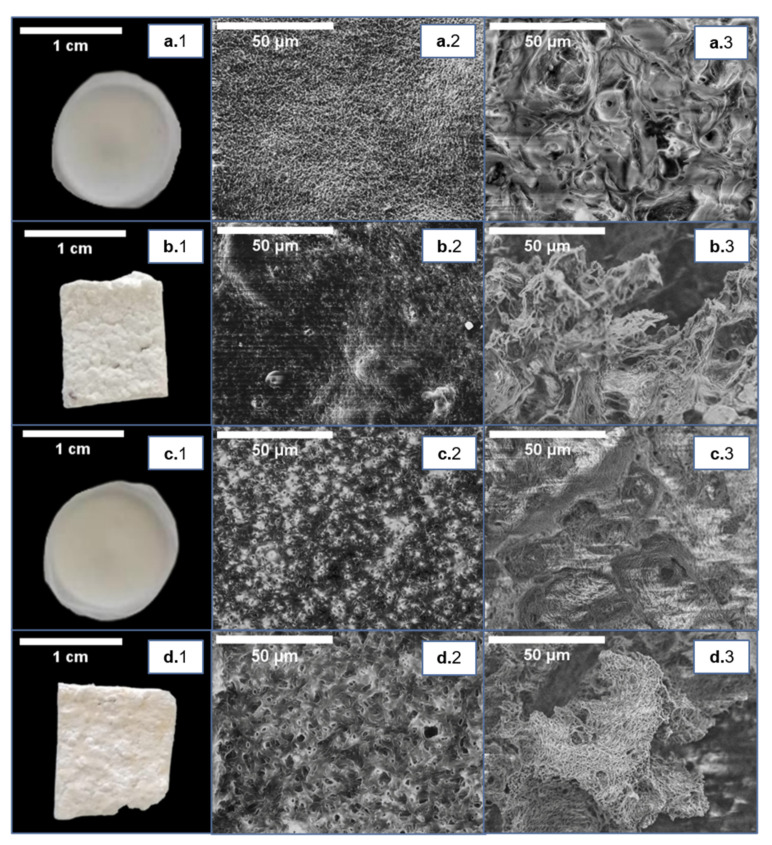
SCPL scaffolds prepared from P(3HB) (**a.1**–**a.3**,**b.1**–**b.3**) and P(3HB-*co*-3HV) (**c.1**–**c.3**,**d.1**–**d.3**), by mixing the porogen (NaCl) with the polymer solution in chloroform (**a.1**,**c.1**) or by pouring the polymer solution over a porogen layer (**b.1**,**d.1**). Surface (**center images**, (**a.2–d.2**)) and cross-section (**right images**, (**a.3–d.3**)) observed by scanning electron microscopy (SEM) analysis.

The first approach (porogen added to the polymer solution) resulted in compact and homogenous scaffolds for both biopolymers (Figure 3). As shown in Figure 3a,c, the scaffolds were white and opaque. The SEM images confirmed the scaffold compactness, and, although P(3HB-*co*-3HV) showed some porosity at its surface and cross-section (Figure 3(c.2,c.3)), reduced porosity was displayed by P(3HB) (Figure 3(a.2,a.3)). Significantly higher porosity was observed for the SCPL scaffolds obtained by the second approach (pouring technique), as shown by the SEM analysis (Figure 3b,d). Moreover, this process facilitated the elimination of the porogen from the polymeric structure since the NaCl crystals are not as tightly packed into the polymer matrix. Thus, the pouring technique was chosen to prepare the SCPL scaffolds for further testing. 

The SCPL P(3HB) and P(3HB-*co*-3HV) scaffolds obtained by the pouring technique disclosed a white, opaque and foam-like aspect (Figure 3(b.1,d.1)), with a thickness of 1.50 ± 0.14 and 1.40 ± 0.08 mm, respectively (Table 2). The P(3HB) scaffolds were highly brittle and cracked easily, while those obtained from P(3HB-*co*-3HV) were slightly more flexible and, thus, less fragile. The SEM analysis showed the scaffolds had irregular surfaces (Figure 3(b.2,d.2)), with a layered porous structure (Figure 3(b.3,d.3)), which may be responsible for their foam-like structure. Similar structures have been reported for P(3HB), P(3HB-*co*-3HV) and P(3HB-*co*-3HHx) scaffolds prepared by the SCPL technique [29,30], as well as for other polymers, such as polylactic acid [31], chitosan/gelatine [32] and polycaprolactone [33].

#### 3.3.2. Water Emulsion Templating

**Figure 4:**
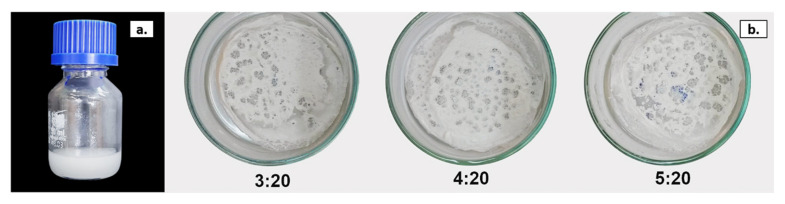
(**a**) Water:P(3HB-*co*-3HV) emulsion (2:20 ratio, *v/v*) and (**b**) P(3HB-*co*-3HV) scaffolds produced with 3:20, 4:20 and 5:20 water:polymer solution ratios (*v/v*).

**Figure 5:**
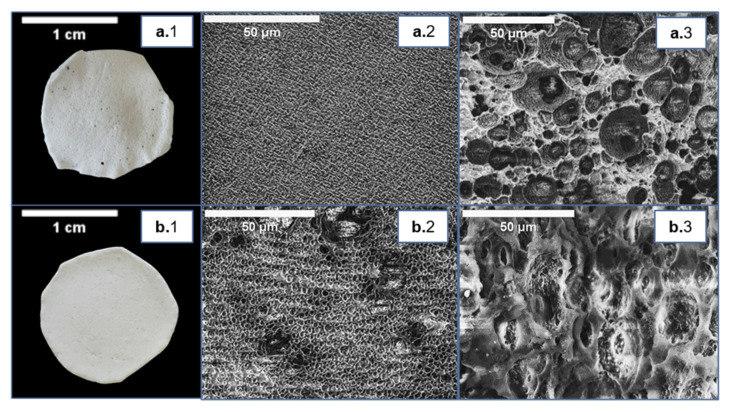
Emulsion-templated scaffolds prepared from P(3HB) (**a.1**) and P(3HB-*co*-3HV) (**b.1**), and surface (**center images**, (**a.2**) and (**b.2**), respectively) and cross-section (**right images**, (**a.3**) and (**b.3**), respectively) obtained by scanning electron microscopy (SEM) analysis.

Water-in-chloroform emulsion templating combined with solvent evaporation was tested as a method to prepare porous scaffolds from P(3HB) and P(3HB-*co*-3HV). The main advantage of this processing technique stems from its simplicity and the fact it is without specialized equipment. To create emulsions, water was slowly added to the polymer solution in chloroform (at a concentration of 4 wt.%) and, upon mixing, a thick, mayonnaise-like mixture was obtained (Figure 4a). Different water:polymer solution ratios were tested (2:20, 3:20, 4:20 and 5:20, *v/v*). The 2:20 ratio produced the most stable emulsions (Figure 4a) for both biopolymers, resulting in homogenous scaffolds (Figure 5). Increasing the water content (3:20, 4:20 and 5:20 ratios) led to unstable emulsions with phase separations between the polymer solution and water and, consequently, to heterogeneous scaffolds with mesh-like macrostructures and holes (Figure 4b). This can be explained by Ostwald ripening, an event where large droplets grow at the expense of smaller ones, which leads to coalescence and eventually emulsion break down [12]. Therefore, the 2:20 ratio was chosen for the fabrication of emulsion-templated scaffolds for further characterization.

The emulsion-templated P(3HB) scaffolds presented a thickness of 0.61 ± 0.13 mm (Table 2) and an opaque white color (Figure 5(a.1)). These scaffolds had a rough surface, as demonstrated by the SEM analysis (Figure 5(a.2)), but no discernible microporosity was displayed, likely due to the rapid evaporation of the solvent from the scaffold. Nonetheless, the cross-section revealed distinguishable porosity, with spherical pores of varying size (0.78–3.58 μm) that appeared to be interconnected (Figure 5(a.3)). 

The emulsion-templated P(3HB-*co*-3HV) scaffolds were also white and opaque (Figure 5(b.1)) and were thinner (0.26 ± 0.023 mm in thickness) than the P(3HB) scaffolds (Table 2). The SEM images showed some porosity on the scaffolds’ surface (Figure 5(b.2)), as well as interconnected pores (Figure 5(b.3)). Nevertheless, the pores’ size was mostly in the 1.35–5.0 μm range, slightly larger than for the emulsion-templated P(3HB) scaffolds.

The optimal pore size for cell culture is highly dependent on the cell and type and can range from 5–400 µm. For example, fibroblasts have an optimum pore size of 5–15 µm and chondrocytes of 70–120 µm, while it is 60–150 µm for vascular smooth muscle cell binding and 100–400 µm for bone regeneration [34]. Our data indicate that for the purpose of cell culture, the pore size of our emulsion-templated scaffolds needs further improvement to increase its dimensions, but might be most suited for fibroblast culture, which requires a smaller pore size.

Analogous porous morphology was reported for emulsion-templated P(3HB) with pore sizes in the 0.5–1.0 μm range [23,24] and P(3HB-*co*-3HV) scaffolds (3.0–7.0 µm pore size) [29]. 

### 3.4. Characterization of the P(3HB) and P(3HB-co-3HV) Porous Scaffolds

#### 3.4.1. Porosity

The SCPL scaffolds based on P(3HB) and P(3HB-*co*-3HV) presented porosity values of 54 ± 4% and 63 ± 3%, respectively (Table 2), whereas those prepared by emulsion-templating had lower porosity values, 27 ± 16% and 49 ± 10%, respectively. These results show that both scaffolding techniques were successful in conferring porosity to the prepared PHA structures. Similar porosity values (45–52%) were reported for P(3HB) scaffolds prepared by emulsion-templating with water [23,24]. Moreover, comparable porosity values were reported for SCPL scaffolds based on P(3HB-*co*-3HV) (37–56%) [35]. SCPL scaffolds produced with other biopolymers showed higher porosities, such as polycaprolactone (PCL) (88%) [33], poly(lactic acid) (PLA) (90.6–93.3%) [36] and poly(lactic-*co*-glycolic acid) (PLGA) (88 ± 2%) [37], due to differences in material properties or/and processing conditions. 

#### 3.4.2. Water Contact Angle

**Figure 6:**
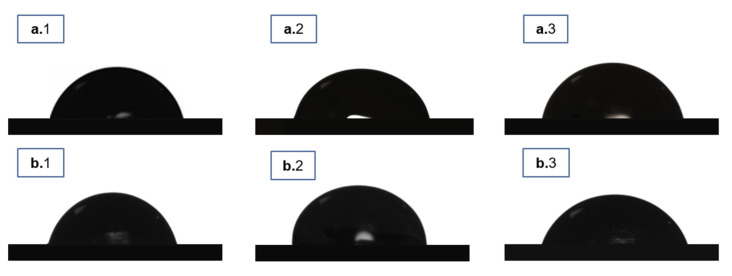
Water droplet images of (**a.1**) P(3HB) films; (**a.2**) SCPL P(3HB) scaffolds; (**a.3**) emulsion-templated P(3HB); (**b.1**) P(3HB-*co*-3HV) films; (**b.2**) SCPL P(3HB-*co*-3HV) scaffolds and (**b.3**) emulsion-templated P(3HB-*co*-3HV).

Water droplet images obtained from water contact angle measurements are represented in Figure 6. For P(3HB), and the water contact angle of the scaffolds was not significantly altered by either scaffolding technique. The SCPL P(3HB) scaffolds presented a contact angle of 72.0 ± 1.2°, which is only slightly lower than the value found for the cast films (81.0 ± 0.8°), while the emulsion-templated P(3HB) scaffolds had a similar value (79.7 ± 0.7°) (Table 2). Similar results were obtained for P(3HB-*co*-3HV) with the tested scaffolding techniques, where SCPL scaffolds displayed a water contact angle of 80.2 ± 1.1° (Table 2), akin to the corresponding cast films (78.0 ± 0.4°), and the emulsion-templated scaffolds displayed a slightly lower value (72.7 ± 0.1°) (Table 2). Values in the range of 65–69° were reported for other polymer-based SCPL scaffolds, including P(3HB) and P(3HB-*co*-3HV) (67.5 ± 1.1° and 65.8 ± 1.3°, respectively), or PLA (68.7°) [31]. For P(3HB-*co*-3HV) emulsion-templated scaffolds, a higher value of 103 ± 5° was reported [29].

#### 3.4.3. Swelling in Water 

Considerable water uptake ability was conferred to P(3HB) and P(3HB-*co*-3HV) structures for both scaffolding techniques, especially by SCPL (Table 2). Compared to the cast films, for which no water absorption was noticed, the SCPL scaffolds displayed swelling degrees of 175.0 ± 0.1% and 171.0 ± 5.2%, for P(3HB) and P(3HB-*co*-3HV), respectively. These values are in the range of those reported for PLA (75–290%) [36], although lower than those reported for PLGA (630%) [37]. 

Although lower swelling degree values were obtained for the emulsion-templated P(3HB) and P(3HB-*co*-3HV) scaffolds (35.3 ± 21% and 36.2 ± 3.7%, respectively) (Table 2), both polymeric structures were able to absorb water. The lower swelling degree compared to the SCPL scaffolds can be correlated with the emulsion-templated scaffolds’ lower porosity (Table 2). The obtained values are higher than those displayed by P(3HB) emulsified with Span 80 (15%) but lower than the values reported for emulsification with water in the presence of lithium sulphate monohydrate in the water phase (65–75%) [24].

### 3.5. Impact of the Scaffolding Techniques on the Biopolymers’ Physical and Chemical Properties 

#### 3.5.1. Molecular Mass Distribution

For both scaffolding techniques, a reduction of the biopolymers’ M_w_ was noticed but the values were kept at the same order of magnitude (Table 1). The PDI remained the same for the emulsion-templated scaffolds, but that of the SCPL scaffolds increased from 1.80 and 1.60 to 2.56 and 2.57, for P(3HB) and P(3HB-*co*-3HV), respectively, indicating the salt leaching procedure impacted the size distribution of the macromolecules. This might be correlated to hydrolysis of the ester bond, especially upon the dissolution of NaCl crystals in the ultrasonic water bath, which may lead to polymer degradation and reduction of molecular weight [38].

#### 3.5.2. Thermal Properties

**Figure 7:**
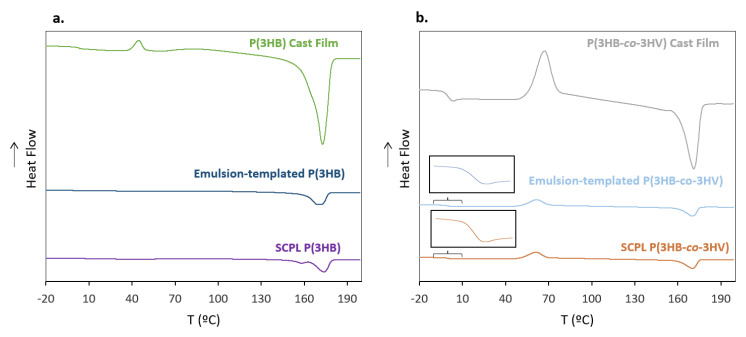
Differential scanning calorimetry (DSC) thermograms for (**a**) P(3HB) and P(3HB)-based scaffolds and (**b**) P(3HB-*co*-3HV) and P(3HB-*co*-3HV)-based scaffolds.

The thermograms of the third heating cycle for the polymers and their corresponding scaffolds are represented in Figure 7, and all three heating cycles are shown in Appendix A. Although no significant changes were noticed in the T_m_ of the biopolymers for either scaffolding technique, their melting enthalpy values were reduced compared to the corresponding cast films (Table 1). For P(3HB), the SCPL scaffolds displayed a melting enthalpy of 44.0 J g^−1^ that correlated to a crystalline fraction of 30.0%, while for the emulsion-templated scaffolds, the melting enthalpy was 49.9 J g^−1^ with a crystalline fraction of 34.2% (Table 1). These values are lower than those of the cast films (76.5 J g^−1^ and 52.4%, respectively) (Table 1). Similar behavior was observed for P(3HB-*co*-3HV) with the SCPL scaffolds presenting a ∆H_m_ of 12.4 J g^−1^ and the emulsion-templated ones a value of 16.5 J g^−1^, compared to 34.5 J g^−1^ for the cast films (Table 1). Concomitantly, the crystalline fraction was also lower for the scaffolds (8.5 and 11.3%), compared to 23.6% for the cast films (Table 1). This behavior has been reported for scaffolds based on P(3HB-*co*-3HV) using SCPL [39] and is correlated to the difference in solvent evaporation conditions that affects the crystallization dynamics experienced by the biopolymer chains during processing. Accordingly, DSC observations therefore support the hypothesis that during both SCPL and emulsion-templating, the mobility of polymer molecules was lower to promote crystal formation during chloroform evaporation, resulting in a decreased degree of crystallinity [40]. In terms of cold crystallization, the phenomena were not observed for either P(3HB)-based scaffold. In contrast, for both SCPL and emulsion-templated P(3HB-*co*-3HV)-based scaffolds, there are clear exothermic peaks related to cold crystallization, with crystallization temperatures of 51 and 50 °C (Table 1), and corresponding cold crystallization enthalpies of 32.8 and 30.8 J g^−1^ (Table 1), respectively. These values are in the range of those obtained for the P(3HB-*co*-3HV) cast film (T_c_ = 56 °C and ∆H_cc_ = 34.7 J g^−1^) (Table 1).

No glass transition could be observed for either P(3HB) scaffolds (Table 1). For P(3HB-*co*-3HV), on the other hand, the SCPL and the emulsion-templated scaffolds exhibited T_g_ values of −0.55 °C and −0.68 °C, respectively (Table 1). These values were slightly lower than the ones observed in the copolymer’s cast films (0.74 °C) (Table 1), and this can be correlated to the lower degree of crystallization of the scaffolds in comparison with the cast film [22]. 

There was no major alteration of the biopolymers’ thermal degradation temperature (T_deg_) upon their processing into the emulsion-templated scaffolds compared to the cast films (Table 1). However, for the SCPL scaffolds, the T_deg_ decreased from 293 °C to 278 °C, for P(3HB), and from 292 °C to 284 °C, for P(3HB-*co*-3HV), which may be related to the observed lowering of Mw, together with an increase of PDI (Table 1), upon processing by the SCPL technique.

### 3.6. Mechanical Properties of the Emulsion-Templated Scaffolds

The SCPL scaffolds were too heterogeneous and fragile to allow for mechanical testing, which might be explained by their higher PDI value (Table 1) and considerably higher porosity (Table 2), compared to the emulsion-templated scaffolds. Therefore, only the emulsion-templated scaffolds were characterized and compared with the cast films. As shown in Table 3, the P(3HB) and P(3HB-*co*-3HV) cast films presented tensile strength at breaking values of 20.80 ± 0.92 MPa and 8.90 ± 0.64 MPa, respectively. The higher value displayed by the P(3HB) cast films can be linked to the biopolymer’s higher crystallinity degree [41]. The obtained values are within the range of those reported for P(3HB) and P(3HB-*co*-3HV) cast films (19.28–26 MPa and 1.22–21.8 MPa, respectively) [41,42,43,44]. 

The P(3HB) and P(3HB-*co*-3HV) emulsion-templated scaffolds presented similar tensile strength at breaking values (3.18 ± 0.19 MPa and 3.35 ± 0.54 MPa, respectively) (Table 3). These values are lower than those of the corresponding cast films, indicating that the applied processing techniques impacted the biopolymer morphology, rendering them less resistant to mechanical stress. The lower tensile strength at breaking values can be explained by the different crystalline arrangements the biopolymer macromolecules adopted after processing [22]. Nevertheless, these values are much higher than emulsion-templated acrylate scaffolds tested for bone tissue engineering (0.11 ± 0.01–2.03 ± 0.33 MPa) [45] but similar to the values presented by fibrin emulsion-templated scaffolds for skin regeneration (4.25 ± 0.63–5.13 ± 0.51 MPa) [46]. 

The P(3HB) and P(3HB-*co*-3HV) scaffolds showed elongation at breaking values of 13.6 ± 0.4% and 14.8 ± 1.7%, respectively (Table 3). These results show that after the emulsion templating processing into scaffolds, both materials exhibit similar responses to deformation. Compared to the corresponding cast films, it can be noticed that the emulsion-templated P(3HB) scaffolds were less ductile than the cast film (20.4 ± 4.21% elongation), while similar resistance to deformation was observed for the P(3HB-*co*-3HV) scaffold and the cast film (13.4 ± 2.1% elongation). The values observed for the emulsion-templated PHA-based scaffolds are among the ones reported for emulsion-templated acrylate-based scaffolds reported for bone tissue engineering (2.60 ± 0.61%–21.86 ± 2.87%) [45].

The emulsion-templated scaffolds displayed Young’s Modulus values considerably lower than the cast films (Table 3). P(3HB) presented a Young’s modulus of 0.07 ± 0.01 MPa and P(3HB-*co*-3HV) a value of 0.11 ± 0.02 MPa, close to the reported for polystyrene-based scaffolds produced through emulsion templating (0.15 ± 0.01 MPa) [47] and silk fibroin emulsion-templated scaffolds (0.228–0.364 MPa) [48], used for tissue engineering, which indicates that the scaffolds produced in this work have adequate properties to serve as scaffolds for tissue engineering purposes.

P(3HB) and P(3HB-*co*-3HV) as scaffold materials showed comparable behavior in terms of surface hydrophilicity and water uptake ability; still, P(3HB-*co*-3HV) scaffolds demonstrated superior porosity and flexibility, as well as pore size, making it the superior material to produce scaffolds intended for cell culture.

The SCPL method led to scaffolds with high porosity and water uptake ability; however, they showed poor mechanical properties with excessive brittleness, making them unsuitable for cell culture. To combat this issue, in further studies this approach can be used in combination with other biocompatible materials, such as polylactic acid (PLA) [36] and polycaprolactone (PCL) [49], to form blends with enhanced mechanical performance. Moreover, the emulsion-templated technique yielded scaffolds with adequate porosity and pore morphology, water uptake ability and mechanical properties for tissue engineering; nonetheless, pore size could be further improved by the incorporation of additives for emulsion stability, such as surfactants (e.g., Span80) or electrolytes (e.g., lithium sulphate monohydrate) [24].

## 4. Conclusions

In this work, SCPL and emulsion templating were employed to prepare porous scaffolds based on the biopolyesters P(3HB) and P(3HB-*co*-3HV). The emulsion-templated structures possessed properties adequate for their use as scaffolds for 3D cell culture, namely, suitable pore morphology and interconnected pores, adequate surface hydrophilicity and water uptake ability, and suitable mechanical properties. Among the two tested biopolymers, P(3HB-*co*-3HV) proved to have superior properties, given its lower crystallinity and higher flexibility. Therefore, implementing emulsion templating for preparing porous P(3HB-*co*-3HV) scaffolds is a viable pathway to produce novel porous scaffolds that might find use in tissue engineering applications.

## Figures and Tables

**Table 1:** Characterization of the P(3HB) and P(3HB-*co*-3HV) scaffolds (SCPL, Solution Casting with Particulate Leaching; M_w_, molecular weight; PDI, polydispersity index; T_g_, glass transition temperature; T_m_, melting temperature; T_deg_, degradation temperature; T_c_, crystallization temperature; X_c_, crystallinity fraction; ΔH_cc_, cold crystallization enthalpy; ΔH_m_, melting enthalpy; SCPL, Solution Casting with Particulate Leaching; n.o., not observed).

Sample	M_w_ (×10^5^ Da)	PDI	T_g_ (°C)	T_m_ (°C)	T_deg_ (°C)	T_C_ (°C)	∆H_cc_ (J g^−1^)	∆H_m_ (J g^−1^)	X_c_ (%)
P(3HB)	5.2	1.8	n.o.	176	293	39	4.4	76.5	52.4
SCPL P(3HB) scaffold	3.6	2.56	n.o.	174	278	n.o	n.o	44	30
Emulsion-templated P(3HB) scaffold	3.8	1.75	n.o.	173	291	n.o	n.o	49.9	34.2
P(3HB-*co*-3HV)	5.6	1.6	0.74	171	292	56	34.7	34.5	23.6
SCPL P(3HB-*co*-3HV) scaffold	3.7	2.57	−0.55	172	284	51	32.8	12.4	8.5
Emulsion-templated P(3HB-*co*-3HV) scaffold	4	1.69	−0.68	172	292	50	30.8	16.5	11.3

**Table 2:** Characterization of the P(3HB) and P(3HB-*co*-3HV) cast films and porous scaffolds in terms of thickness, porosity, water contact angle and swelling in water (SCPL, Solution Casting with Particulate Leaching; n.o., not-observed).

Biopolymer	Scaffold	Thickness (mm)	Porosity (%)	Contact Angle (θ)	Swelling (%)
P(3HB)	Cast film	0.16 ± 0.01	n.o.	81.0 ± 0.8	0
	SCPL	1.50 ± 0.14	54 ± 4	72.0 ± 1.2	175.0 ± 0.1
	Emulsion	0.61 ± 0.13	27 ± 16	79.7 ± 0.7	35.3 ± 21.0
P(3HB-*co*-3HV)	Cast film	0.19 ± 0.02	n.o.	78.0 ± 0.4	0
	SCPL	1.40 ± 0.08	63 ± 3	80.2 ± 1.1	171.0 ± 5.2
	Emulsion	0.26 ± 0.02	49 ± 10	72.7 ± 0.1	36.2 ± 3.7

**Table 3:** Mechanical properties of the cast films and the porous scaffolds obtained by the emulsion-templating technique.

Biopolymer	Scaffold	Tensile Strength (MPa)	Elongation at Breaking (%)	Young’s Modulus (MPa)
P(3HB)	Cast film	20.8 ± 0.92	20.4 ± 4.21	2.18 ± 0.08
	Emulsion	3.18 ± 0.19	13.6 ± 0.44	0.07 ± 0.01
P(3HB-*co*-3HV)	Cast film	8.90 ± 0.64	13.4 ± 2.12	1.38 ± 0.86
	Emulsion	3.35 ± 0.54	14.8 ± 1.74	0.11 ± 0.02

**Figure A1:**
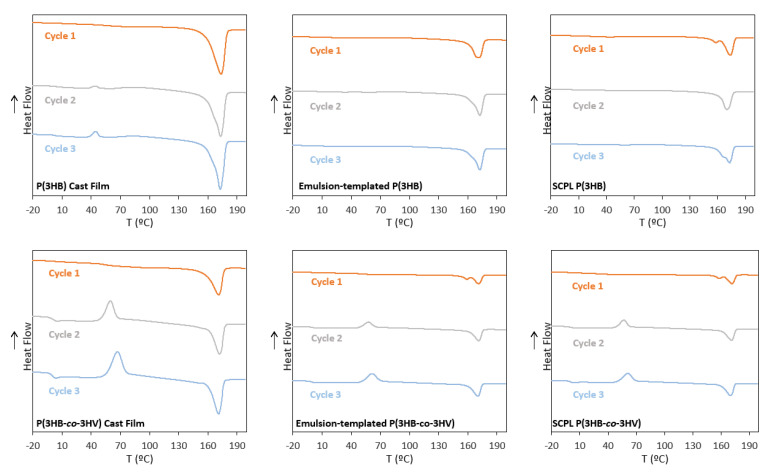
Differential scanning calorimetry (DSC) thermograms of heating cycles 1, 2 and 3 for P(3HB) and P(3HB)-based scaffolds, as well as P(3HB-*co*-3HV) and P(3HB-*co*-3HV)-based scaffolds.

## Data Availability

Data will be made available upon request.
